# Repurposing Dihydropyridines for Treatment of *Helicobacter pylori* Infection

**DOI:** 10.3390/pharmaceutics11120681

**Published:** 2019-12-15

**Authors:** Andrés González, Javier Casado, Eduardo Chueca, Sandra Salillas, Adrián Velázquez-Campoy, Vladimir Espinosa Angarica, Lucie Bénejat, Jérome Guignard, Alban Giese, Javier Sancho, Philippe Lehours, Ángel Lanas

**Affiliations:** 1 Aragon Institute for Health Research (IIS Aragón), San Juan Bosco 13, 50009 Zaragoza, Spain; 2 Institute for Biocomputation and Physics of Complex Systems (BIFI), Mariano Esquilor (Edif. I+D), 50018 Zaragoza, Spain; 3 Department of Biochemistry and Molecular & Cellular Biology, University of Zaragoza, Pedro Cerbuna 12, 50009 Zaragoza, Spain; 4 Centro de Investigación Biomédica en Red de Enfermedades Hepáticas y Digestivas (CIBERehd), Monforte de Lemos 3-5, 28029 Madrid, Spain; 5 ARAID Foundation, Government of Aragon, Ranillas 1-D, 50018 Zaragoza, Spain; 6 Cancer Science Institute, National University of Singapore, 14 Medical Drive, #12-01, Singapore 117599, Singapore; 7 UMR1053 Bordeaux Research in Translational Oncology, INSERM, Université Bordeaux, BaRITOn, 33000 Bordeaux, France; 8 French National Reference Center for Campylobacters & Helicobacters, 33000 Bordeaux, France; 9 Digestive Diseases Service, University Clinic Hospital Lozano Blesa; San Juan Bosco 15, 50009 Zaragoza, Spain; 10 Department of Medicine, Psychiatry and Dermatology, University of Zaragoza, Pedro Cerbuna 12, 50009 Zaragoza, Spain

**Keywords:** repurposing, *Helicobacter pylori*, dihydropyridines, HsrA

## Abstract

Antibiotic resistance is a major cause of the increasing failures in the current eradication therapies against *Helicobacter pylori*. In this scenario, repurposing drugs could be a valuable strategy to fast-track novel antimicrobial agents. In the present study, we analyzed the inhibitory capability of 1,4-dihydropyridine (DHP) antihypertensive drugs on the essential function of the *H. pylori* response regulator HsrA and investigated both the in vitro antimicrobial activities and the in vivo efficacy of DHP treatments against *H. pylori*. Six different commercially available and highly prescribed DHP drugs—namely, Nifedipine, Nicardipine, Nisoldipine, Nimodipine, Nitrendipine, and Lercanidipine—noticeably inhibited the DNA binding activity of HsrA and exhibited potent bactericidal activities against both metronidazole- and clarithromycin-resistant strains of *H. pylori*, with minimal inhibitory concentration (MIC) values in the range of 4 to 32 mg/L. The dynamics of the decline in the bacterial counts at 2 × MIC appeared to be correlated with the lipophilicity of the drugs, suggesting different translocation efficiencies of DHPs across the bacterial membrane. Oral treatments with 100 mg/kg/day of marketed formulations of Nimodipine or Nitrendipine in combination with omeprazole significantly reduced the *H. pylori* gastric colonization in mice. The results presented here support a novel therapeutic solution for treatment of antibiotic-resistant *H. pylori* infections.

## 1. Introduction

*Helicobacter pylori* is a microaerophilic Gram-negative bacterium that currently colonizes the gastric epithelium of almost 4.4 billion people worldwide [1]. Unless treated, infection of human stomach usually persists lifelong, causing gastric inflammation and contributing to the pathogenesis of peptic ulcer disease, gastric adenocarcinoma, and mucosa-associated lymphoid-tissue (MALT) lymphoma [2,3]. *H. pylori* has been classified as a class I human carcinogen by the International Agency for Research of Cancer. In fact, near 90% of non-cardia gastric cancer worldwide and approximately 5% of the total burden from all cancers globally can be attributable to *H. pylori* infection [4].

The increasing prevalence of antibiotic-resistant strains of *H pylori*, the high rates of antibiotic-associated side effects and the low patient compliance have led to a significant reduction in the eradication rates of traditional standard triple therapies, which are based on the co-administration of a proton pump inhibitor (PPI) and two antibiotics, clarithromycin and either amoxicillin or metronidazole for 7 to 10 days. To face this emerging health issue, the current guidelines recommend a quadruple therapy (PPI + amoxicillin + metronidazole + clarithromycin) administered for 14 days as first-line strategy in adults, while the PPI triple therapy has been restricted to areas with known low clarithromycin resistance [5]. Further, in February 2017 the World Health Organization included *H. pylori* in its first ever list of antibiotic-resistant “priority pathogens”, a catalogue of 12 families of bacteria that pose at present the greatest threat to human health [6]. Nowadays, an effective novel therapy against *H. pylori* is mandatory in order to overcome the current resistome and minimize side effects on normal microbiota.

One of the major causes of the present antibiotic resistance crisis is the lack of development of new antimicrobial drugs by the pharmaceutical industry due to reduced economic incentives and challenging regulatory requirements. Since any new antibiotic is reserved in clinical practice for only the worst cases of illness due to the fear of promoting drug resistance, its reduced use and relatively low cost for the patient represent a diminished return on investment [7,8]. Drug repurposing has emerged as an alternative approach for the development of novel and effective antimicrobial therapies. This strategy, consisting in discovering a novel clinical use for an existing drug previously approved for a different therapeutic indication, can minimize the costs and risks associated with drug development programs and accelerate the delivery of new therapeutics to patients with refractory infections or emerging infectious diseases [9,10]. Hence, the search for drug repurposing candidates able to inhibit the growth of pathogens by acting specifically on new molecular targets can be a valuable route for drug discovery.

In *H. pylori*, the essential response regulator HsrA appears to be a promising target for drug development [11,12,13,14,15,16]. HsrA (for homeostatic stress regulator) is unique among members of the *Epsilonproteobacteria* [12] and functions as a global transcriptional regulator, synchronizing metabolic functions and virulence with the availability of nutrients and cell division [13], mediating also the response to oxidative stress [14]. Attempts at both deletion and overexpression of HsrA have been unsuccessful, supporting not only an essential function of the regulator but also a very tight post-transcriptional control of its expression that ensures appropriate levels of the protein into the cell [12,15]. In a previous study, we screened the Prestwick Chemical Library^®^, a collection of 1120 FDA-approved, off-patent small molecules for identifying compounds that specifically bind to HsrA and potentially inhibit its essential function. At least 14 compounds (1.25%) of the Prestwick repurposing library bound to the native state of HsrA and notably increased the protein conformational stability against thermal denaturation, causing a shift of the protein unfolding curve to higher temperatures due to the increased melting temperature (*T*_m_) of the protein–compound complexes [17]. Despite most of the identified HsrA binders from the Prestwick library consisting of naturally occurring flavonoids, several other FDA-approved drugs formed stable complexes with HsrA and enhanced its thermodynamic stability, including the 1,4-dihydropyridine (DHP) calcium channel blocker Nicardipine [18,19]. In the present study, we investigated the effect of Nicardipine and other commercially available DHP derivative drugs on the biological activity of the *H. pylori* essential response regulator HsrA and characterized the molecular interaction between these drugs and the target protein. Bactericidal activities of selected DHP-class inhibitors of HsrA as well as their potential synergistic effects in combination with conventional antibiotics used as first-line treatment against *H. pylori* infection (metronidazole and clarithromycin) were also evaluated. In addition, the efficacy of two representative DHP drugs in eradicating the *H. pylori* gastric mucosal colonization was assessed in a mouse model.

## 2. Materials and Methods 

### 2.1. Chemicals

DHP drugs for in vitro experiments were purchased from Sigma-Aldrich (Saint Louis, MO, USA) and properly stored according to the manufacturer’s instructions. Stock solutions of each drug were freshly prepared at 20 mM in 100% dimethyl sulfoxide (DMSO) for electrophoretic mobility shift assays and isothermal titration calorimetry analyses, and at 10.24 g/L in 100% DMSO for minimal inhibitory concentration (MIC)/minimal bactericidal concentration (MBC) determinations. Since DHPs are light-sensitive compounds, all stock solutions were protected from light. Metronidazole and clarithromycin were obtained from Sigma-Aldrich. Stock solutions of these antibiotics in 100% DMSO were prepared at 10.24 g/L and stored at −20 °C for up to 30 days. Marketed formulations (oral tablets) of DHP drugs for in vivo efficacy studies were purchased from STADA S.L. (Bad Vilbel, Germany).

### 2.2. Bacterial Strains, Culture Media and Growth Conditions 

*H. pylori* reference strains ATCC 700392, ATCC 43504 (metronidazole-resistant), and ATCC 700684 (clarithromycin resistant) were purchased from the American Type Culture Collection and used in the in vitro antibacterial assays. The strains were grown in Blood Agar Base No.2 (OXOID) supplemented with 8% defibrinated horse blood (OXOID) in a humidified microaerobic incubator (85% N_2_, 10% CO*_2_*, 5% O_2_) at 37 °C for 48–72 h. For certain experiments, bacteria were grown for 48–72 h at 37 °C in brain heart infusion broth (OXOID) supplemented with 4% fetal bovine serum (Gibco). For in vivo experiments, the CagA^+^ mouse colonizing strain pre-mouse Sydney Strain 1 (PMSS1) [20,21], obtained from the French National Reference Center for Campylobacters & Helicobacters (www.cnrch.fr), was used. For infection inocula, *H. pylori* PMSS1 was grown on in-house selective Wilkins Chalgren agar plates [22] under microaerobic conditions and transferred to Brucella broth medium as described below.

### 2.3. Electrophoretic Mobility Shift Assays 

The in vitro biological activity of the *H. pylori* HsrA response regulator and its potential inhibition by DHP drugs were assessed by electrophoretic mobility shift assay (EMSA) as previously described [17]. Briefly, 120 ng of target DNA (promoter region of the *H. pylori porGDAB* operon [14]) was mixed with 6 µM of recombinant HsrA [17] in the presence of increasing concentrations (0.1 to 2 mM) of selected DHP drugs. Mixtures of DNA and protein in a 20 μL reaction volume containing 10 mM bis-Tris (pH 7.5), 40 mM KCl, 100 mg/L BSA, 1 mM DTT and 5% glycerol were incubated at room temperature for 20 min and then separated by 6% native polyacrylamide gel electrophoresis. A 150 bp fragment of the *Anabaena* sp. PCC 7120 gene *pkn22* was included as non-specific competitor DNA in all assays. EMSA gels were stained with SYBR Safe^®^ (Thermo Fisher Scientific, Bothell, WA, USA) and analyzed using the Bio-Rad Gel Doc 2000 imaging system.

### 2.4. Minimal Inhibitory and Bactericidal Concentrations

MIC determinations were carried out by the broth microdilution method as previously described [17,23,24]. Briefly, *H. pylori* strains were grown for 48 h at 37 °C under microaerobic conditions (85% N_2_, 10% CO_2_, 5% O_2_) in brain heart infusion broth supplemented with 4% fetal bovine serum (BHI+FBS). Fresh inoculums at ~10^6^ CFU/mL were prepared by dilution of the bacterial cultures in the same BHI+FBS medium to a final optical density at 600 nm of 0.01. DHP drugs were serially two-fold diluted in the bacterial inoculum from 256 to 0.125 mg/L using sterile 96-well flat-bottom microtiter plates and then incubated under microaerobic conditions at 37 °C for 72 h. For MBC determinations, 10 μL aliquots of two dilutions around the MIC value were plated on inhibitor-free Blood Agar Base No.2 with 8% defibrinated horse blood and incubated for 72 h. Each experiment was performed twice in triplicate

### 2.5. Time–kill Kinetics Assays

Time–kill curves for selected DHP drugs were determined using the *H. pylori* strain ATCC 700684 [17]. A bacterial suspension of 5.0 × 10^5^ CFU/mL was freshly prepared in BHI+FBS and mixed with each DHP at a final concentration of 2-fold the corresponding MIC value. DMSO instead of drugs was included as control. Colony forming units (CFUs) were determined after 4, 8, and 24 h of drug exposition by culturing on Blood Agar Base No.2 supplemented with 8% defibrinated horse blood for 72 h under microaerobic conditions. Each determination was performed twice in triplicate and the results were presented as log_10_ CFU/mL versus incubation time. Statistical analysis was performed using the Mann–Whitney U test. A *p* value smaller than 0.05 was considered significant.

### 2.6. Checkerboard Assays

Antibacterial synergies of selected DHPs in combination with first-line anti-*H. pylori* antibiotics were tested by the checkerboard assay [17]. Using 96-well flat-bottom microtiter plates, we assayed the antibacterial effect of the interaction of a range of 2-fold dilutions of clarithromycin or metronidazole with a range of 2-fold dilutions of each selected DHP. Serial dilutions of both antimicrobials (antibiotic and DHP) were firstly prepared using two different sterile microtiter plates, one compound was diluted along the rows in a first plate, and the other compound was diluted along the columns of a second plate. Then, both gradients were mixed in a third microtiter plate and inoculated with a freshly prepared bacterial suspension of *H. pylori* at 2 × 10^6^ CFU/mL in BHI+FBS. Plates were incubated for 48 h at 37 °C under microaerobic conditions. After this time, microbial growth was colorimetric revealed by addition of 0.01 mg/mL resazurin (Sigma-Aldrich). The interaction between drugs was determined by calculating the fractional inhibitory concentration index (FICI) as: FIC_A_ (MIC_A_ in the presence of B/MIC_A_ alone) + FIC_B_ (MIC_B_ in the presence of A/MIC_B_ alone) [25].

### 2.7. Isothermal Titration Calorimetry Assays

Thermodynamic parameters of the molecular interaction between HsrA and its DHP-class inhibitors were analyzed by isothermal titration calorimetry (ITC) using an AutoiTC200 calorimeter (MicroCal, Malvern Instruments, Malvern, Worcestershire, UK). A solution of 20 µM of the protein in 50 mM Tris-HCl [pH 8], 150 mM NaCl, 10% glycerol, 1% DMSO was titrated with 200 µM of the corresponding ligand (DHP) dissolved in the same buffer at 25 °C. A total of 19 injections of 2 μL was added sequentially to the sample cell after 150 s spacing to ensure that the thermal power returned to the baseline before the next injection [26]. Dissociation constants and binding enthalpies were calculated by non-linear least squares regression data analysis using the software package Origin 7.0 (OriginLab, Northampton, MA, USA).

### 2.8. Molecular Docking 

Molecular docking analyses were performed using the program AutoDock Vina 1.1.2 [27] in order to estimate the conformation of the HsrA-DHP complexes. The chemical structures of ligands (Nifedipine, Nicardipine, Nisoldipine, Nimodipine, Nitrendipine, and Lercanidipine) were retrieved from the PubChem database (https://pubchem.ncbi.nlm.nih.gov/). The structure of the *H. pylori* HsrA response regulator (2HQR, model 1, chain A) was downloaded from the Protein Data Bank (https://www.rcsb.org/). The protein and the ligands were prepared using the AutoDock Tools program. Rotatable bonds were defined as free for the ligands and rigid for the protein. The AutoGrid algorithm was used to estimate the interaction energy of each ligand pose. The pose that exhibited the lowest free energy of interaction (ΔG, kcal/mol) for each ligand was considered as its predicted model of binding to the target protein.

### 2.9. Mouse Infection and DHP Treatment 

The in vivo evaluation of anti-*Helicobacter pylori* activity of selected DHPs was carried out in the C57BL/6 mouse model [20]. The experiments were performed in specific pathogen-free animal facilities at the University of Bordeaux (France). The study was approved by a local ethical committee of the University of Bordeaux and conforms to the French Ministry of Agriculture Guidelines on Animal Care and the French Committee of Genetic Engineering, with respect to the principle of the 3Rs (Replacement, Reduction and Refinement; approval number 201812051729186–V6 APAFiS #17999). 

Six-week-old specific pathogen-free C57BL/6 female mice (*n* = 38) were purchased from Charles River Laboratories (Saint-Germain-Nuelles, France). Animals were housed in polycarbonate cages and acclimatized for one week before starting the experiments. Two groups of 9 mice were fasted to facilitate bacterial colonization and then force-fed for 3 consecutive days with a dose of 10^8^ CFU in 100 μL of *H. pylori* strain PMSS1 [21,28]. Bacterial inocula were freshly prepared in Brucella broth medium from cultures grown on in-house Wilkins Chalgren agar plates supplemented with 10% human blood and 10 µg/mL of vancomycin, 5 µg/mL of trimethoprim, 1 µg/mL of amphotericin B and 2 µg/mL of cefsulodin [22] under microaerobic conditions (85% N_2_, 10% CO_2_, 5% O_2_) at 35 °C for 48 h. 

Four weeks after inoculation, all mice of each group were treated orally with 100 mg/kg/day of marketed formulations (oral tablets, STADA S.L., Bad Vilbel, Germany) of Nimodipine or Nitrendipine in combination with omeprazole (140 mg/kg/day) daily, for 7 days. Two additional groups of 10 mice were used as controls, one non-infected and one *H. pylori*-infected non-treated group. Both control groups received omeprazole (140 mg/kg/day) for the 7 days of treatments. 

All the animals were sacrificed by cervical dislocation one month after the end of treatments. The stomach of each mouse was properly isolated, opened by the large curvature using a small curved chisel, and put in a petri dish with saline buffer to remove the food inside. The organs were cut along the axis of the large curvature, and then cut along the axis of the small curvature. A fragment of the right half-stomach cleared of the cardia was introduced into an RNase/DNase free tube containing 200 μL of sterile saline buffer for bacteriological cultures and molecular studies.

### 2.10. Bacterial Counts and qPCR

Quantitative cultures to determine the bacterial load in gastric biopsies were carried out as follows. Stomach fragments from each mouse were harvested as described above and weighed. Then, the stomach fragments were crushed using a sterile pestle and serial dilutions of the homogenized organs were spread on Wilkins Chalgren agar plates supplemented with 10% human blood and 10 µg/mL of vancomycin, 5 µg/mL of trimethoprim, 1 µg/mL of amphotericin B and 2 µg/mL of cefsulodin. Plates were incubated under microaerobic conditions (85% N_2_, 10% CO_2_, 5% O_2_) at 35 °C and colonies were counted after at least 5 days of incubation. *H. pylori* was identified by its phenotypic and biochemical characteristics (morphology, urease test, oxidase test). Colony counting was performed by two independent experiments and the results were expressed as CFU/mg of stomach.

The efficacy of the DHP treatments to eradicate *H. pylori* stomach colonization in the mouse model was also determined by quantitative PCR. DNA was extracted from each homogenized stomach using the Arrow system (Nordiag, Bergen, Norway). A quantitative PCR using Fluorescence Resonance Energy Transfer (FRET) technology targeting DNA coding for *H. pylori* 23S ribosomal RNA (rRNA) was performed. Primers described by Oleastro and co-workers [29] were used to amplify the 23S rRNA gene and primers described by Laur and co-workers [30] were used to amplify the glyceraldehyde 3-phosphate dehydrogenase (GAPDH) gene. Each target was tested in duplicate on all samples. A standard curve was prepared using serial dilutions of a DNA extract from a CFU/mL calibrated bacterial suspension of the *H. pylori* PMSS1 strain. The LightCycler^®^ 480 SYBR^®^ Green I Master Mix (Roche Diagnostics, Rotkreuz, Switzerland), compatible with the LightCycler^®^ 480 thermocycler (Roche Diagnostics), was used according to the manufacturer’s instructions. The SYBR^®^ Premix Ex Taq™ Mix (Tli RNaseH Plus) (Takara Bio Inc., Shiga, Japan) compatible with the PCR thermocycler CFX96™ (Bio-Rad Laboratories, Hercules, CA, USA) available at the TBMCore real-time PCR platform (University of Bordeaux, France) was used according to the manufacturer’s instructions. The PCR started with a 95 °C DNA denaturation step for 3 min, followed by 40 cycles comprising two steps: a 95 °C denaturation step for 5 s and a 60 °C primer hybridization step for 30 s. After each cycle, fluorescence was measured in order to quantify newly synthesized DNA. At the end of the procedure, a melting curve was generated by a slow elevation in the temperature from 65 to 95 °C and the continuous measurement of fluorescence. The generation of this melting curve permitted the verification of one specific peak at the expected melting temperature for each product, which showed the PCR specificity. The final results were expressed as a ratio of bacteria/murine cells. DNA extracted from the m-ICcl2 murine epithelial cell line available in the laboratory was used to express the results as a ratio of bacteria/murine cells. The detection limit of this method was around 0.001 bacteria/murine cell for *H. pylori*, as previously described [28]. 

### 2.11. Statistical Analysis 

Bacterial counts (CFU/mg) and qPCR results (*Helicobacter*/10,000 murine cells) were compared between each DHP-treated group and the infected non-treated control group. Statistical analyses were carried out using the non-parametric Mann–Whitney test. A *p* value smaller than 0.05 was considered significant. All statistics were performed using GraphPad Prism 6.0 (GraphPad Software, Inc., La Jolla, CA, USA).

## 3. Results

### 3.1. DHPs Inhibit the DNA Binding Activity of the H. pylori Essential Response Regulator HsrA

Previous high-throughput screening of the Prestwick Chemical Library^®^ using a fluorescence-based thermal shift assay identified Nicardipine (Cardene) as a specific HsrA binder that preferentially bound to the native state of the *H. pylori* essential response regulator and increased the protein conformational stability, causing a shift of the protein unfolding curve to higher temperatures [17]. Since Nicardipine shares a similar chemical structure with other DHP-class calcium channel blockers, we investigated the effect of Nicardipine and other five commercially available DHP derivative drugs (Table 1) on the in vitro biological activity of HsrA. 

**Figure 1:**
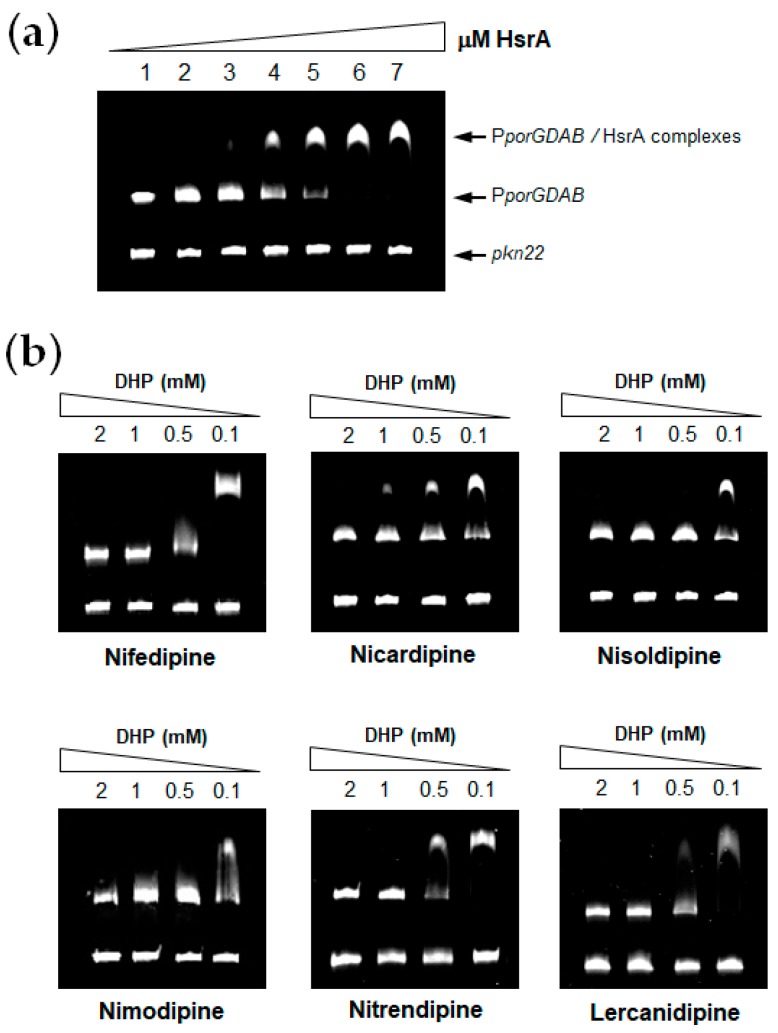
In vitro inhibition of HsrA DNA binding activity by selected DHP drugs. (**a**) Electrophoretic mobility shift assays (EMSAs) showing the ability of recombinant HsrA to specifically bind the promoter region of target *porGDAB* operon. Increasing concentrations of HsrA (indicated in μM) were mixed with 120 ng of target promoter and separated on a 6% PAGE. The *Anabaena* gene *pkn22* was included as non-specific competitor DNA in all assays. (**b**) DNA fragments were mixed with 6 μM of recombinant HsrA protein in the presence of 2, 1, 0.5 and 0.1 mM of DHPs.

HsrA functions as a transcriptional activator of gene expression; hence, it interacts with DNA and binds to specific sequences located in target promoters, thereby modulating gene expression. EMSA analyses of HsrA in the presence of its target promoter P*_porGDAB_* demonstrated a high affinity of HsrA by its target DNA in a concentration-dependent manner (Figure 1a). Under the experimental conditions used in EMSAs, 120 ng of target DNA were completely and specifically complexed to HsrA from 6 μM of the recombinant protein. Hence, this concentration was subsequently used for EMSA inhibition assays in the presence of 100 µM to 2 mM of each DHPs. As shown in Figure 1b, all the DHPs tested notably inhibited the in vitro DNA binding activity of HsrA to its target promoter. No relevant differences were observed in the inhibitory capacities of Nifedipine, Nicardipine, Nisoldipine, Nimodipine, Nitrendipine and Lercanidipine on the HsrA biological activity according to EMSAs. In all cases, 1 mM of DHP was sufficient to completely inhibit the in vitro biological activity of 6 μM of recombinant HsrA protein. In the cases of Nicardipine, Nisoldipine and Nifedipine, the activity of the regulator was partially inhibited even at 100 μM of these drugs under the experimental conditions used in our binding assays. 

### 3.2. Analysis of the Molecular Interaction between HsrA and its DHP-Class Inhibitors 

**Figure 2:**
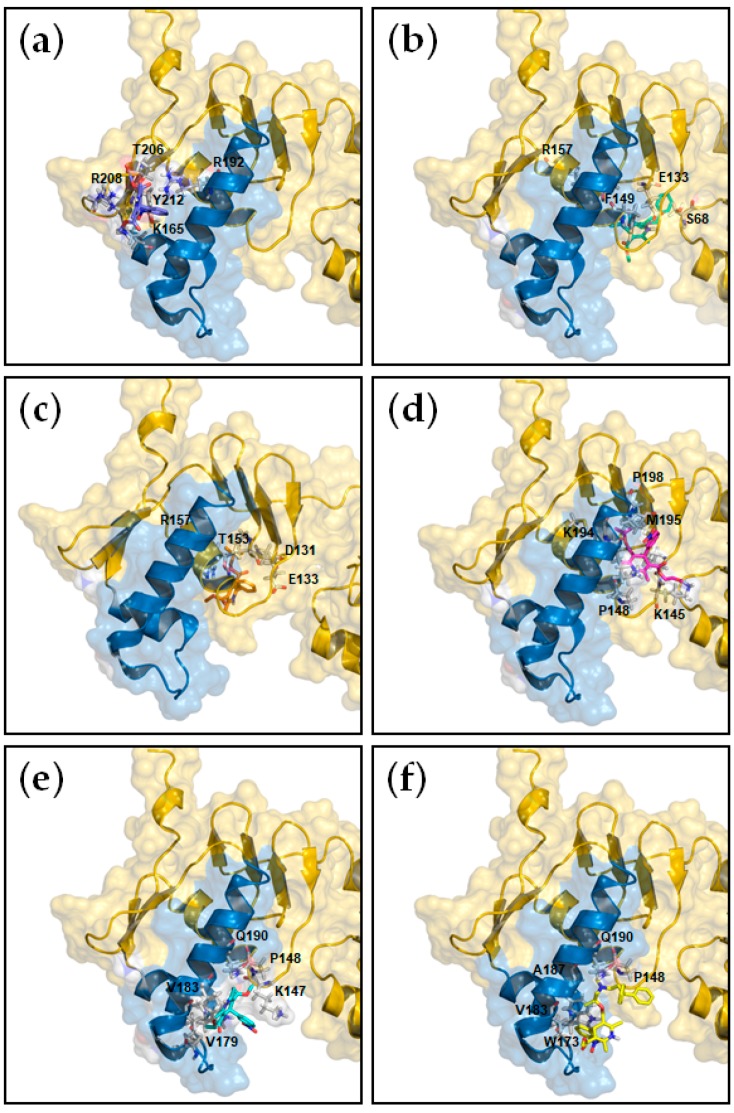
Local overviews of the best ranked docking poses of Nifedipine (**a**), Nicardipine (**b**), Nisoldipine (**c**), Nimodipine (**d**), Nitrendipine (**e**) and Lercanidipine (**f**) interaction with HsrA. Ribbon model and transparent molecular surface showing the interacting residues of HsrA to each DHP. The helix-turn-helix (HTH) DNA binding motif of HsrA is highlighted in blue. Some interacting residues are indicated.

In order to determine the affinity, the enthalpy and the stoichiometry of binding as well as to unravel the structural basis of the interaction between HsrA and its DHP-class inhibitors, ITC measurements and molecular docking analyses were carried out. As shown in Table 2, the ITC data provided dissociation constants in the micromolar range in all cases, while complexes of a 1:1 stoichiometry were observed for all DHPs (Figure S1, Supplementary Materials), indicating that each HsrA monomer of the dimeric protein binds one molecule of DHP. Molecular docking suggested that DHP inhibitors bind to the native conformation of HsrA preferably at the C-terminal effector domain, interacting with amino acid residues directly involved in the structure of the helix-turn-helix (HTH) DNA binding motif, but also with other residues presumably essential for the domain stabilization (Table 2, Figure 2).

### 3.3. DHP-Class Inhibitors of HsrA Exhibit Strong Bactericidal Activities against H. pylori

DHP-class inhibitors of HsrA were evaluated for their antimicrobial properties against three different strains of *H. pylori*, including two reference strains resistant to metronidazole (ATCC 43504), and clarithromycin (ATCC 700684). As shown in Table 3, Nifedipine, Nicardipine, Nisoldipine, Nimodipine, Nitrendipine and Lercanidipine showed potent bactericidal activities against *H. pylori*. The minimal inhibitory concentration (MIC) values of all tested DHPs were in the range of 4 to 32 mg/L, while most of these antihypertensive drugs exhibited minimal bactericidal concentration (MBC) values against *H. pylori* ≤ 16 mg/L. Despite no relevant differences observed in the anti-*H. pylori* activities of this set of DHP drugs according to their MIC or MBC values, Lercanidipine appeared slightly less effective on the metronidazole-resistant strain.

**Figure 3:**
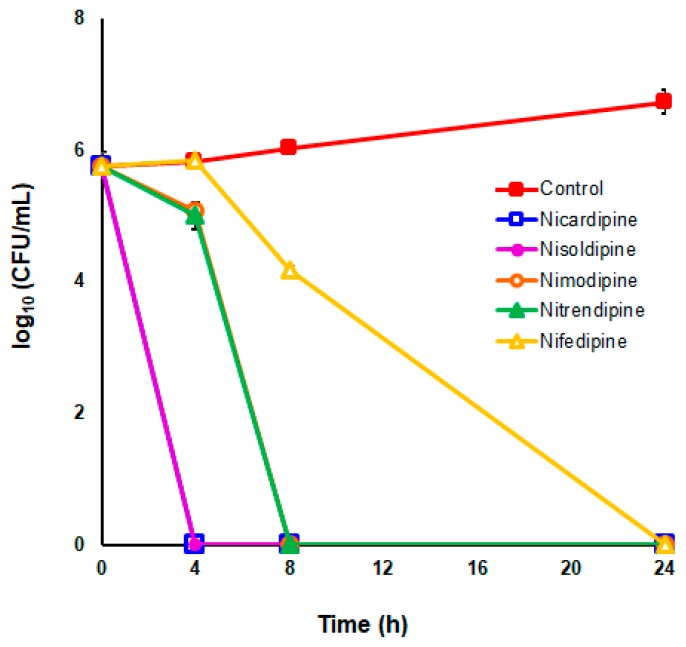
Time–kill kinetics of DHP drugs against *H. pylori* strain ATCC 700684. Bacterial counts were determined at time zero and after 4, 8, and 24 h of incubation with two times the MIC. Mixtures of bacteria with DMSO (vehicle) instead of DHP were used as controls. Values are the averages of six independent determinations; vertical bars represent standard deviations. Please note that in some instances, the error bar is smaller than the symbols used.

To further analyze the bactericidal effect of DHP drugs against *H. pylori*, time–kill kinetics were carried out at 2 × the MIC values for the five selected most potent DHPs against the *H. pylori* strain ATCC 700684. As depicted in Figure 3, no live bacteria could be detected at 24 h after exposition to each DHP at these concentrations; however, significant differences were observed in the time–kill kinetics caused by each drug. Notably, Nicardipine and Nisoldipine were completely lethal even after only 4 h of exposition to these drugs. Nevertheless, no killing effect was perceived at this time with the exposition to 2 × MIC of Nifedipine. In fact, the decline in bacterial counts due to the exposition to Nifedipine was significantly slower (*p* < 0.05) compared to those produced by the rest of bactericidal DHPs.

### 3.4. Combinatory Effects of DHPs with Metronidazole and Clarithromycin against Antibiotic-Resistant H. pylori Strains

The in vitro interactions between bactericidal DHPs and metronidazole or clarithromycin were assessed by the checkerboard assay. In order to best appreciate the effect of each antimicrobial combination on the improvement of the antibiotic efficacy, the tests were performed using the corresponding antibiotic-resistant *H. pylori* strain, either the metronidazole-resistant strain ATCC 43504 (MIC = 64 mg/L) or the clarithromycin-resistant strain ATCC 700684 (MIC = 16 mg/L). As shown in Table 4, DHPs exhibited only additive effect (slight synergy) or no interactions with the two conventional antibiotics tested. However, in some cases, the increases observed in the activities of the antibiotics in combination with certain DHPs were sufficient to change the classification of the corresponding strain from resistant to susceptible, taking into account the breakpoints recommended for each antibiotic [32]. Hence, Nimodipine reduced the MIC value of metronidazole up to 8 times (FIC = 0.125) in the metronidazole-resistant strain ATCC 43504, while Lercanidipine reduced up to 16 times the MIC value of clarithromycin (FIC = 0.0625) in the clarithromycin-resistant strain ATCC 700684.

### 3.5. Nimodipine and Nitrendipine Significantly Reduced the H. pylori Gastric Colonization in Mice 

**Figure 4:**
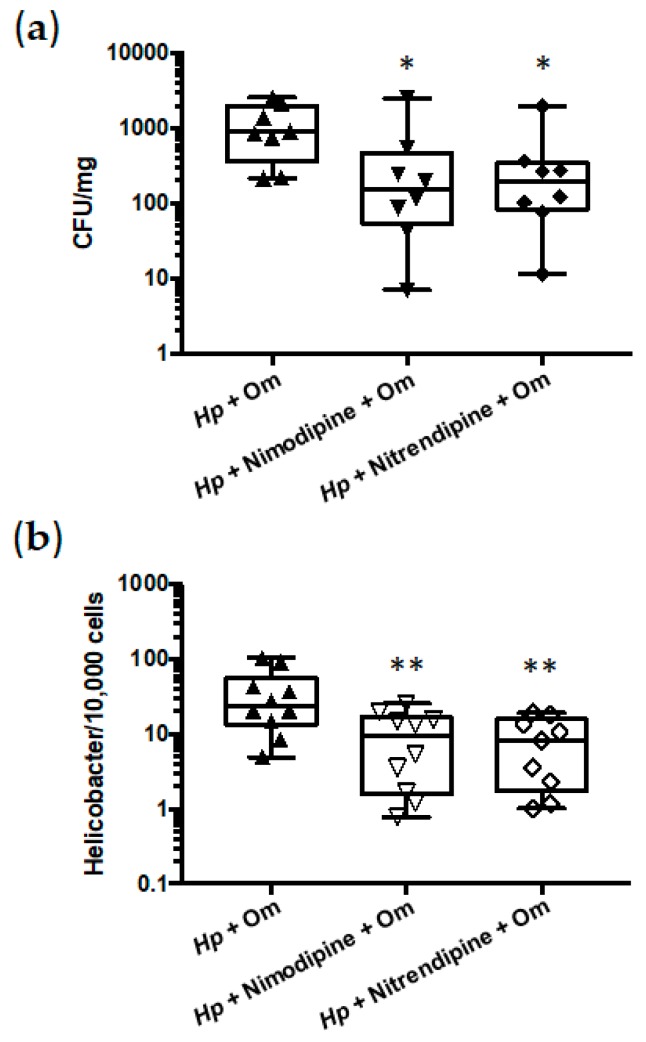
Antimicrobial effects of Nimodipine and Nitrendipine against *H. pylori* stomach colonization in mice. (**a**) Bacterial counts from gastric biopsies are presented as CFU of *H. pylori* per mg of stomach. (**b**) Quantitative PCR from gastric biopsies are presented as a ratio of bacteria per 10,000 murine cells. *Hp*, *H. pylori* strain pre-mouse Sydney Strain 1 (PMSS1). Om, omeprazole. *Hp* + Om, infected non-treated mice (*n* = 10), *Hp* + Nimodipine + Om (*n* = 9) and *Hp* + Nitendipine + Om (*n* = 9). Graphic representations are box plots, with the box representing 50% of values around the median (horizontal line) and the whiskers representing the minimum and maximum of all the data. * *p* < 0.05, ** *p* < 0.01 *H. pylori*-infected non-treated mice versus *H. pylori*-infected treated mice.

Since DHPs exhibited potent in vitro antimicrobial activities against *H. pylori*, their therapeutic efficacies in vivo were determined in the mouse model of gastric colonization. Given the poor bioavailability of DHPs due to their low solubility and substantial first-pass elimination [33], we selected for the in vivo experiments, two DHPs that exhibit high oral LD_50_ values in mice—Nimodipine and Nitrendipine (Table 1)—which allowed for treating the animals with higher therapeutic doses. Thus, after four weeks of infection, mice were treated orally with 100 mg/kg/day of Nimodipine (9.4 times lower than LD_50_) or Nitrendipine (25.4 times lower than LD_50_) in combination with omeprazole for 7 days. Both antimicrobial therapies led to significant reduction in the *H. pylori* colonization of mice stomachs compared to those observed in non-treated animals, as determined by two different approaches (Figure 4).

## 4. Discussion

Developing a new chemical entity drug and delivering it to the market, known as *de novo* drug discovery, is a time-consuming and expensive process with increasing regulatory requirements and a high risk of failure—all of which could make the pharmaceutical industry a less desirable choice for investors. Nowadays, drug repurposing has become a successful strategy to fast-track therapeutic agents for the treatment of several emerging or rare diseases, such as AIDS, Alzheimer’s disease and cancer [10,34,35,36,37,38,39,40], but also infections with multidrug-resistant strains of clinically relevant pathogens [9,41,42,43,44]. 

Calcium channel blockers (CCBs) are small molecules that preferentially interact with L-type voltage-operated calcium channels expressed in skeletal, smooth and cardiac muscles. These drugs inhibit the influx of calcium into the sarcoplasm and thereby reduce contraction of arterial smooth muscle and myocardium, leading to a decrement in blood pressure [18]. Among CCBs, DHPs are the most frequently prescribed antihypertensive drugs worldwide given the strong antihypertensive effects, safety, long-half-life and greater efficacy of some of these drugs and the minimization of side effects (arterial hypotension, headache, flushing and ankle edema) with long-acting formulations [19]. Beyond their efficacy in the control of hypertension and their demonstrated capacity to reduce cardiovascular morbidity and mortality, DHPs have shown strong antimicrobial activities against some bacterial and fungal pathogens such as *Listeria monocytogenes* [45], *Mycobacterium tuberculosis* [46], *Staphylococcus aureus* [47], *Escherichia coli* [48], *Pseudomonas aeruginosa* [49], *Salmonella typhimurium* [50], *Aspergillus fumigatus* and *Candida albicans* [51]. In addition, DHP drugs have shown antiprotozoal activity against *Leishmania* spp. [52,53] and *Trypanosoma cruzi* [53,54].

*H. pylori* is the most prevalent human pathogen worldwide and a major cause of gastric and duodenal ulcers and gastric cancer. The increasing resistance to first-line antibiotic drugs, especially metronidazole and clarithromycin [55,56], has had a dramatic impact on the eradication rates, which have fallen to 70% in the last few years [1]. In order to overcome the antimicrobial resistance strategies evolved by this pathogen, novel molecular targets have emerged as candidates for therapeutic interventions [23,24,57,58,59,60,61]. In a previous work, we validated a new effective anti-*H. pylori* therapeutic target, the essential response regulator HsrA [17]. Several FDA-approved small-molecule drugs, including natural flavonoids such as apigenin, chrysin, kaempferol and hesperetin, noticeably inhibited the biological activity of HsrA and exhibited strong bactericidal activities against different strains of *H. pylori*, including both metronidazole- and clarithromycin-resistant strains. In the present study, we demonstrated that commercially available and highly prescribed DHP drugs such as Nifedipine, Nicardipine, Nisoldipine, Nimodipine, Nitrendipine and Lercanidipine strongly inhibited the in vitro biological activity of HsrA by interacting with this response regulator preferably by its C-terminal effector domain, as previously observed with natural flavonoids [17]. Therefore, the inhibition of HsrA activity by DHPs could be the result of direct blockage of key amino acid residues involved in the structure of the HTH DNA binding motif and/or conformational changes in the effector domain that destabilize the regulator interaction with its DNA target promoters. The molecular mechanisms responsible for the previously documented antimicrobial activities of DHPs are poorly understood. In *M. tuberculosis*, the antibacterial activity exhibited by DHPs has been related to calcium homeostasis perturbation and loss of the Ca^2+^-dependent DNA gyrase activity [47,62]. Taking into account the results presented here, the antimicrobial activity of DHPs goes beyond their function as calcium channel blockers and additionally, these small-molecule drugs could act as potent inhibitors of essential targets in the microbial cell. In fact, the antiprotozoal effect of DHPs on *T. cruzi* and *Leishmania* sp. has been associated to respiratory chain inhibition [52,54], while no correlation was found between the leishmanicidal activity and the Ca^2+^ channel blocking action of DHPs [52].

All the DHP-class inhibitors of HsrA studied in this work exhibited potent bactericidal activities against different strains of *H. pylori*, including both metronidazole- and clarithromycin-resistant bacteria. No relevant differences were observed in the antimicrobial potency of the six 1,4-DHP derivative drugs according to their MIC or MBC values. However, the dynamics of the decline in the bacterial counts at 2 × MIC appeared to be correlated with the lipophilicity of the drugs. Thus, Nicardipine and Nisoldipine, which show higher log *p* values (lipophilicity parameter) [63], reduced significantly faster the viability of *H. pylori* cells compared to Nifedipine. Correlation between DHP lipophilicity and its antibacterial activity has been previously suggested [47]. Hence, lower lipophilicity of the drug seems to result in poor translocation across the bacterial membrane and consequently lower antimicrobial activity. 

Despite some DHP drugs (e.g., amlodipine and lacidipine) having been previously found to synergistically enhance the efficacy of conventional antibiotics [64,65], none of the six 1,4-DHP derivatives studied here exhibited actual synergy in combination with metronidazole or clarithromycin. However, some additive interactions noticeably reduced the MIC values of these antibiotics against their respective *H. pylori*-resistant strains. Thus, as occurs with other non-antibiotics compounds such as flavonoids, DHPs could reverse antibiotic resistance in certain *H.* *pylori* infections and enhance the action of current antibiotic drugs in novel combinatory therapies. 

In order to best simulate the human pathogenesis of *H. pylori* in the C57BL/6 mouse model, we used the CagA^+^ mouse colonizing strain pre-mouse Sydney Strain 1 (PMSS1) for the in vivo efficacy studies. The PMSS1 model persistently colonizes mice but also mimics the human host response to CagA^+^ *H. pylori* infection, which results in more severe gastritis and a higher risk of gastric adenocarcinoma than the infection with CagA^−^ strains [20,66]. Oral treatments of PMSS1-infected mice with 100 mg/kg/day of marketed formulations of Nimodipine or Nitrendipine in combination with omeprazole (140 mg/kg/day), daily for 7 days, significantly reduced the *H. pylori* gastric colonization of the animal model according to two different methods of evaluation, bacterial counts and qPCR analysis. The DHP oral dose applied to mice in this efficacy study (100 mg/kg/day) corresponds to a human equivalent dose of 8 mg/kg/day [67], the recommended oral dose for Nimodipine (60 mg every 4 h) which could be prescribed to a young adult weighting 45 kg to prevent cerebral vasospasm after aneurysmal subarachnoid hemorrhage [68]. However, the efficacy of these and other DHP derivative drugs as anti-*H. pylori* antimicrobials could be presumably increased if drug bioavailability could be improved, for example, through new formulations via nanotechnology [33] and/or rational chemical modifications of the drug [69]. 

The DHP class of CCBs comprises several tens of low-molecular-weight compounds. Even a very restricted and randomly selected set of six DHP derivatives studied in this work exhibited potent bactericidal activities against antibiotic-resistant strains of *H. pylori,* and at least two of these drugs significantly reduced the *H. pylori* stomach colonization in mice. Novel in vitro and in vivo analyses must be carried out in order to identify the best choice of DHP-class anti-*H. pylori* drug, taking into account several criteria including a high anti-*H. pylori* efficacy, low side effects in humans and a desirable synergy with conventional antibiotics. Despite the fact that DHPs are well-tolerated drugs with a low rate of adverse effects, especially the long-acting formulations [19], the prescription of these anti-hypertensive drugs as antimicrobials should be taken into consideration in terms of their intrinsic vasodilatation action and their potential hypotensive effect in both hypertensive and non-hypertensive patients. Hence, DHPs as novel anti-*H. pylori* drugs could be used as part of personalized therapies where the clinical characteristics of patients must be considered. On the other hand, the currently prescribed DHP CCBs could be employed as “lead compounds” to synthesize more efficacious anti-*H. pylori* drugs, even when the antihypertensive effects of these molecules are mitigated.

## 5. Conclusions

The results presented here support the use of DHP antihypertensive drugs in novel antimicrobial strategies against *H. pylori* infections. Because of their high antimicrobial activities and their potential to reverse antibiotic resistance in certain refractory infections, 1,4-DHP derivative drugs should be included in both preclinical and clinical evaluations of novel and personalized combinatory therapies against *H. pylori.* Further efforts to improve the bioavailability of these drugs as novel antimicrobials must be conducted in order to increase efficacy, reduce doses and mitigate potential side effects.

## 6. Patents

The authors declare that a patent has been filed concerning the use of 4-phenyldihydropyridine derivatives for the treatment and/or prevention of *H. pylori* infection.

## Figures and Tables

**Table 1:** The 1,4-dihydropyridine (DHP) derivative drugs tested in this work as novel antimicrobials against *Helicobacter pylori* infection.

Drug (Brand Name)	DHP Generation	Molecular Formula	Chemical Structure	Mouse LD_50_ (mg/kg, oral) *
Nifedipine (Adalat)	first	C_17_H_18_N_2_O_6_	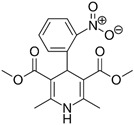	202
Nicardipine (Cardene)	second	C_26_H_29_N_3_O_6_	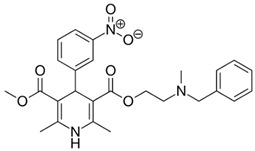	305
Nisoldipine (Sular)	second	C_20_H_24_N_2_O_6_	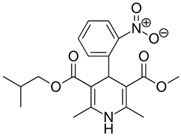	411
Nimodipine (Nimotop)	second	C_21_H_26_N_2_O_7_	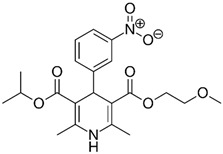	940
Nitrendipine (Baypress)	third	C_18_H_20_N_2_O_6_	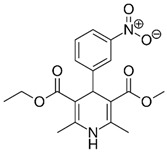	2540
Lercanidipine (Zanidip)	fourth	C_36_H_41_N_3_O_6_	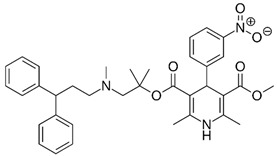	622

* Median lethal dose (LD_50_) data from PubChem. Lercanidipine LD_50_ information from [31].

**Table 2:** Analyses of interaction between HsrA and selected DHP drugs.

DHP Drug	ITC ^a^				Molecular Docking ^b^
	*n*	*K*_d_ (µM)	ΔH (kcal/mol)	ΔG (kcal/mol)	Interacting Residues
Nifedipine	0.74	15	−5.0	−6.6	**T206, R192, Y212, R208, K165**
Nicardipine	0.72	3.0	−2.0	−7.5	T153, E133, F149, R157, S68
Nisoldipine	0.70	11	−2.2	−6.8	R157, T153, E133, P130, D131
Nimodipine	0.71	4.1	−2.1	−7.3	**P198, M195**, I135, P148, K145, **K194**, V144
Nitrendipine	0.75	9.0	−2.6	−6.9	P148, K147, **Q190, V186, V183, V179, W173**
Lercanidipine	0.81	20	−4.8	−6.4	P148, **A187, Q190, V183, W173**

^a^ Absolute error in n is 0.06, relative error in *K*_d_ is 40%, absolute error in ΔH is 0.4 kcal/mol, and absolute error in ΔG is 0.2 kcal/mol. ^b^ Amino acid residues directly involved in forming the helix-turn-helix (HTH) DNA binding motif of HsrA are highlighted in bold fonts.

**Table 3:** Minimal inhibitory and bactericidal concentrations of DHP-class inhibitors of HsrA against different strains of *H. pylori.*

DHP Drug	MIC (MBC), mg/L		
	ATCC 700392	ATCC 43504 (MTZ-R)	ATCC 700684 (CLR-R)
Nifedipine	8 (8)	16 (16)	8 (8)
Nicardipine	8 (8)	8 (8)	8 (8)
Nisoldipine	4 (4)	16 (16)	4 (4)
Nimodipine	8 (8)	16 (32)	4 (4)
Nitrendipine	8 (8)	16 (16)	8 (8)
Lercanidipine	8 (8)	32 (64)	8 (8)
Metronidazole	1 (2)	64 (128)	1 (2)
Clarithromycin	≤0.12 (≤0.12)	≤0.12 (≤0.12)	16 (32)

MTZ-R, metronidazole-resistant strain. CLR-R, clarithromycin-resistant strain. MBC, minimal bactericidal concentration. MIC, minimal inhibitory concentration.

**Table 4:** Combinatory effect of DHPs with metronidazole and clarithromycin against two *H. pylori*-resistant strains.

Strain	Combination Tested	FIC_antibiotic_	FIC_DHP_	FICI *^a^*	Interaction *^b^*
ATCC 43504 (MTZ-R)	MTZ + Nifedipine	0.25	0.5	0.75	additive
	MTZ + Nicardipine	1	1	2	no interaction
	MTZ + Nisoldipine	0.5	0.5	1	additive
	MTZ + Nimodipine	0.125	0.5	0.62	additive
	MTZ + Nitrendipine	1	1	2	no interaction
	MTZ + Lercanidipine	1	1	2	no interaction
ATCC 700684 (CLR-R)	CLR + Nifedipine	0.5	0.5	1	additive
	CLR + Nicardipine	0.25	0.5	0.75	additive
	CLR + Nisoldipine	0.5	0.5	1	additive
	CLR + Nimodipine	0.5	0.5	1	additive
	CLR + Nitrendipine	1	1	2	no interaction
	CLR + Lercanidipine	0.0625	0.5	0.56	additive

*^a^* Fractional inhibitory concentration index (FICI) could be calculated as: FIC_A_ (MIC_A_ in the presence of B/MIC_A_ alone) + FIC_B_ (MIC_B_ in the presence of A/MIC_B_ alone). *^b^* According to the FICI value, the interaction between two compounds against a particular bacterial strain can be classified as: synergism (FICI ≤ 0.5), additive effect (0.5 < FICI ≤ 1), no interaction or neutral (1 < FICI ≤ 4), or antagonism (FICI > 4).

## Supplementary Materials

The following are available online at https://www.mdpi.com/1999-4923/11/12/681/s1. Figure S1: Isothermal titration calorimetry experiments for the interaction of *H. pylori* HsrA response regulator with its DHP-class inhibitors Nifedipine (a), Nicardipine (b), Nisoldipine (c), Nimodipine (d), Nitrendipine (e), and Lercanidipine (f). In the figure, upper panels show the ITC thermograms while lower panels show the binding isotherms.
