# Supplementary material for: Repurposing Dihydropyridines for Treatment of Helicobacter pylori Infection
Source: Pharmaceutics. 2019 Dec 15;11(12):681. doi: 10.3390/pharmaceutics11120681 (PMC6969910; doi:10.3390/pharmaceutics11120681)
Supplement: Supplementary file 1 [file pharmaceutics-11-00681-s001.pdf]

Supplementary Materials: Repurposing Dihydropyridines for Treatment of *Helicobacter pylori* Infection

Andrés González, Javier Casado, Eduardo Chueca, Sandra Salillas, Adrián Velázquez-Campoy, Vladimir Espinosa Angarica, Lucie Bénejat, Jérome Guignard, Alban Giese, Javier Sancho, Philippe Lehours and Ángel Lanas

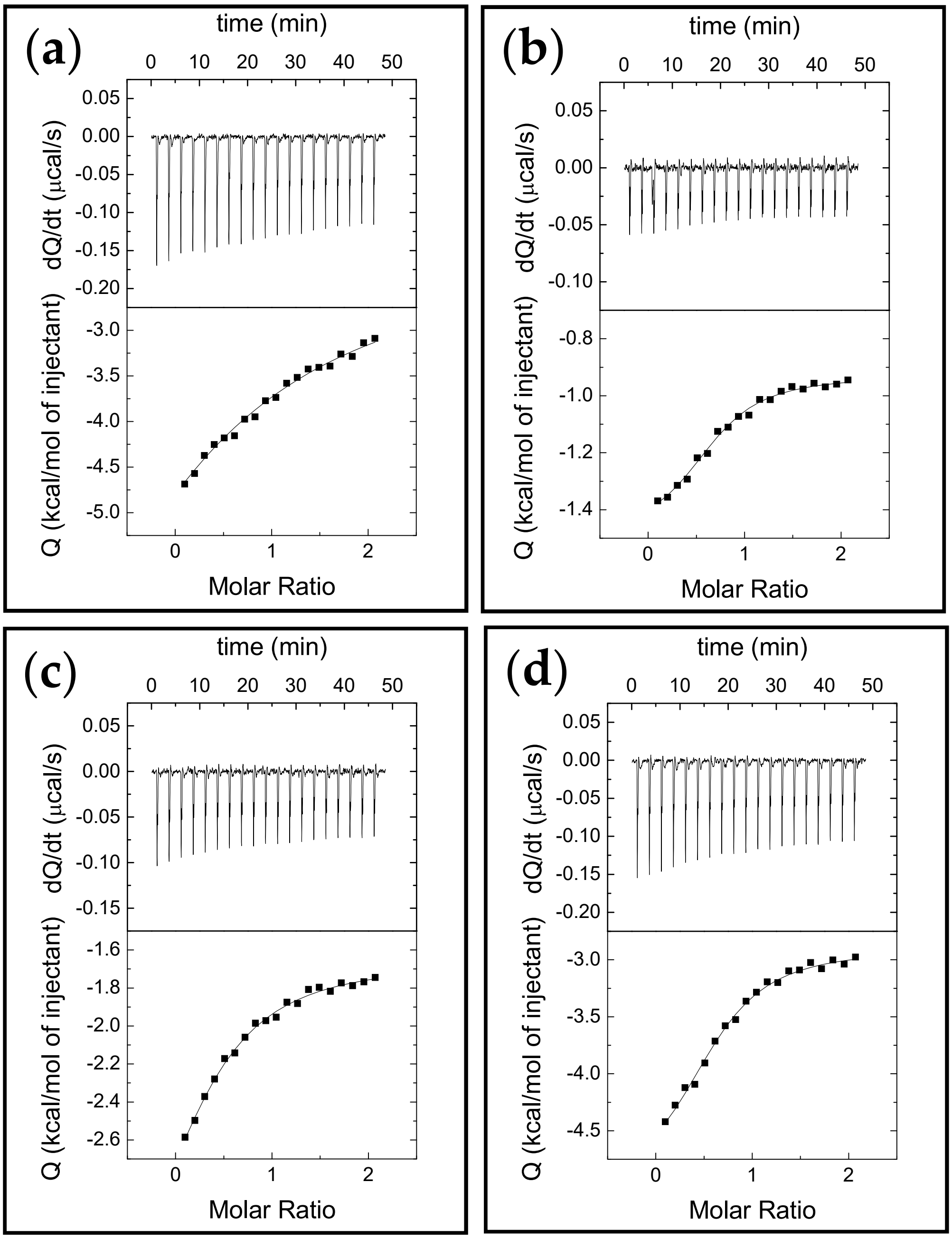

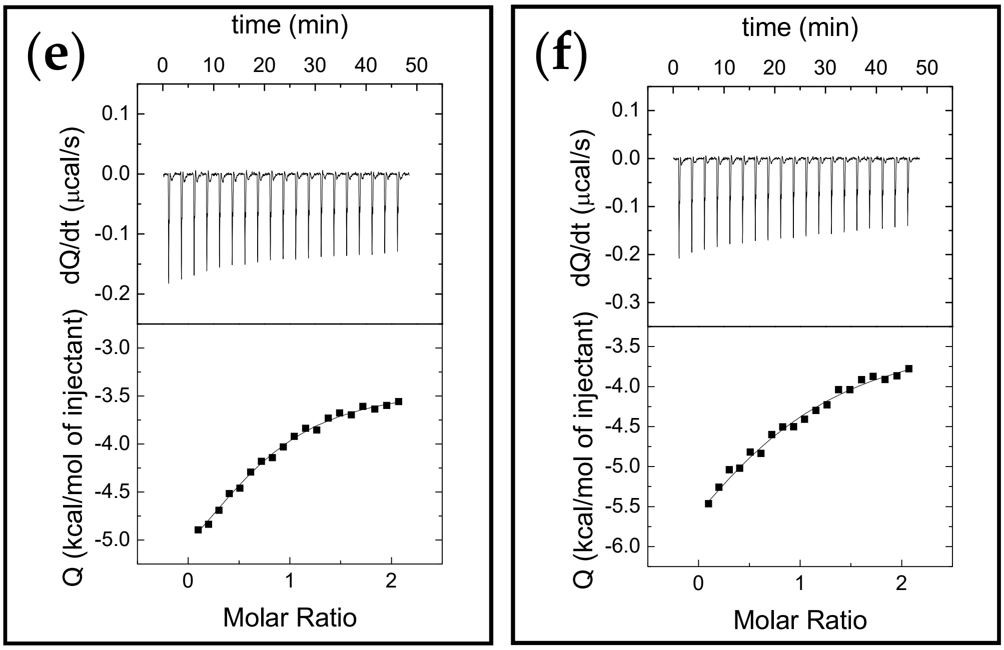

Figure S1. Isothermal titration calorimetry experiments for the interaction of *H. pylori* HsrA response regulator with its DHP-class inhibitors Nifedipine (**a**), Nicardipine (**b**), Nisoldipine (**c**), Nimodipine (**d**), Nitrendipine (**e**), and Lercanidipine (**f**). In the figure, upper panels show the ITC thermograms while lower panels show the binding isotherms.
